# Widespread genetic connectivity of feral pigeons across the Northeastern megacity

**DOI:** 10.1111/eva.12972

**Published:** 2020-04-23

**Authors:** Elizabeth Carlen, Jason Munshi‐South

**Affiliations:** ^1^ Department of Biology Fordham University Bronx NY USA

**Keywords:** *Columba livia*, Northeastern megacity, pigeon, population genetics, Rock Dove, urban evolution

## Abstract

Urbanization may restrict, facilitate, or have no effect on gene flow, depending on the organism and extent of urbanization. In human commensals, with high dispersal ability, urbanization can facilitate gene flow by providing continuous suitable habitat across a wide range. Additionally, suburban or rural areas with lower human population density may act as a barrier to gene flow for these human commensals. Spatial population genetic approaches provide a means to understand genetic connectivity across geographically expansive areas that encompass multiple metropolitan areas. Here, we examined the spatial genetic patterns of feral pigeons (*Columba livia*) living in cities in the eastern United States. We focused our sampling on the Northeastern megacity, which is a region covering six large cities (Boston, Providence, New York City, Philadelphia, Baltimore, and Washington, DC). We performed ddRAD‐Seqon 473 samples, recovered 35,200 SNPs, and then used multiple evolutionary clustering analyses to investigate population structuring. These analyses revealed that pigeons formed two genetic clusters—a northern cluster containing samples from Boston and Providence and a southern cluster containing all other samples. This substructuring is possibly due to reduced urbanization across coastal Connecticut that separates Boston and Providence from New York and mid‐Atlantic cities. We found that pairs of pigeons within 25 km are highly related (Mantel *r* = 0.217, *p* = .001) and that beyond 50 km, pigeons are no more related than they would be at random. Our analysis detected higher‐than‐expected gene flow under an isolation by distance model within each city. We conclude that the extreme urbanization characteristic of the Northeastern megacity is likely facilitating gene flow in feral pigeons.

## 1 INTRODUCTION

Habitat heterogeneity created by landscape features may restrict or aid the ability of an individual to disperse through their environment and between populations. Spatial patterns of genetic variation often directly reflect the landscape composition, providing insight into the specific habitat features that are necessary for gene flow. Habitats that facilitate movement, and thus gene flow, are more likely to sustain genetic diversity and the ability of populations to adapt to evolutionary change (Lande, 1988; Slatkin, 1987). However, if genetic connectivity is limited or restricted, then genetic drift may lead to decreased genetic diversity, increased inbreeding potential, and reduced reproductive success and survival. Ultimately, spatial patterns of genetic diversity in wildlife populations are the outcome of individuals' needs and abilities to move across the landscape. Urbanization, which drastically alters resource availability and habitat, has been repeatedly shown to influence the spatial genetic patterns of wild populations (Combs, Puckett, Richardson, Mims, & Munshi‐South, 2018; Miles, Johnson, Dyer, & Verrelli, 2018; Munshi‐South, Zolnik, & Harris, 2016). In urban areas, fragmentation of natural habitat is typically predicted to reduce gene flow leading to genetic drift and subsequent population differentiation, that is, “urban fragmentation” (reviewed in Miles, Rivkin, Johnson, Munshi‐South, & Verrelli, 2019). However, depending on the dispersal ability of an organism and the habitat heterogeneity between cities, urbanization may also facilitate gene flow leading to lower genetic differentiation between cities, that is, “urban facilitation” (Miles et al., 2019).

Whether an organism is more likely to experience urban fragmentation or urban facilitation is influenced by its life history strategy. Animals that are successful in cities are often dependent on resources provided by humans. These species are generally termed “anthrodependent” (Hulme‐Beaman, Dobney, Cucchi, & Searle, 2016) or “human commensals” (Johnson & Munshi‐South, 2017). Human commensals not only exploit the urban environment but may be obligately dependent upon urban habitats and resources, becoming more successful within cities with few individuals able to live outside of urban areas. These species are also likely to undergo human‐mediated dispersal, with humans intentionally or unintentionally transporting individuals between urban areas, thereby facilitating gene flow and linking populations across cities. Moreover, because commensal organisms rely on anthropogenic resources, their spatial genetic patterns are partially a result of the way humans modify, build, and use cities. Understanding the spatial genetic structure within a human commensal population can provide insights into how social structure, movement, and contact between humans and commensal populations shape the ecological interactions, reproductive dynamics, and pathogen transmission of urban wildlife (Robinson, Samuel, Lopez, & Shelton, 2012).

Feral pigeons (also known as rock doves, *Columba livia*) are a common human commensal found in cities around the world. Pigeons were first brought to North America in the 17th century by French and English colonizers settling in Nova Scotia, Quebec, Massachusetts, and Virginia (Schorger, 1952) with feral populations forming as domestic individuals escaped. In the four centuries since their introduction to North America, pigeons have established themselves in every major city along the Eastern seaboard (eBird, 2012). Pigeons have a longer‐range dispersal potential compared to other urban birds (e.g., house sparrows) and commensals (e.g., rats, bed bugs); however, limited research has been conducted on population genetics in urban pigeons and on gene flow across continuous urban habitat for any species (Jacob, Prévot‐Julliard, & Baudry, 2015; Tang, Low, Lim, Gwee, & Rheindt, 2018). Additionally, the spatial genetic structure of pigeons may be partially shaped by the social policies that guide cities. For example, cities differ in their regulations governing feeding wildlife and their policies behind waste disposal which is a common food resource for urban wildlife. The Northeastern megacity, spanning from Boston, Massachusetts to Washington, DC, is an ideal region to investigate feral pigeon population genetics due to multiple large cities in close geographic proximity to each other. This region consists of multiple metropolitan areas with large amounts of impervious surface and high human population density (urban cores) connected by hamlets and towns with less impervious surface and lower human population density (US Census Bureau, 2012).

Depending on the life history strategies and dispersal abilities of an organism, the Northeastern megacity could be considered a single continuous habitat or multiple distinct habitats. Urban commensals that can traverse larger distances may be able to bypass less‐suitable (i.e., more rural) habitat, whereas species with shorter dispersal ranges are more likely to be confined to city limits (isolation by barrier). Pigeons are capable of traversing the entire distance of the Northeastern megacity in a single day, though the probability of moving this distance within a day, or even within a lifetime, is low (Johnston & Janiga, 1995). Moreover, it is unclear how local variation in habitat quality and resource availability, which fluctuates between municipalities, contributes to movement decisions and the spatial genetic structure of commensal wildlife. In urban commensals, urbanization may facilitate dispersal leading to panmixia or lead to a pattern of isolation by distance due to natural constraints on dispersal distances. Variation in the habitat across the Northeastern megacity could also result in discrete genetic clusters due to urban fragmentation. Across the landscape, nonurban areas may act as a complete barrier to gene flow for urban commensals leading to isolation by barrier. Behavior, physical barriers, and landscape resistance to movement can also create genetic differences among groups of urban animals that range from weak (Adams, van Heezik, Dickinson, & Robertson, 2014; Combs, Byers, et al., 2018; Combs, Puckett, et al., 2018; Hofmeister, Werner, & Lovette, 2019; Tang, Sadanandan, & Rheindt, 2015; Tang et al., 2018) to strong (Gortat, Rutkowski, Gryczynska‐Siemiatkowska, Kozakiewicz, & Kozakiewicz, 2013; Harris et al., 2016; Serieys, Lea, Pollinger, Riley, & Wayne, 2015). Jacob et al. (2015) used microsatellites to detect dispersal and found within‐city dispersal to be common but between‐city dispersal to be a rare event. Currently, it is unknown how pigeons will move through extensive (~750 km), nearly continuous urban habitat.

Resource availability undoubtedly shapes where organisms are found. Pigeons do not migrate seasonally, but their daily movements can vary widely as they travel from nesting to feeding sites. Studies have documented as little as 0.34 kilometers and up to 20 kilometers travelled by pigeons to feed (reviewed in Rose, Nagel, & Haag‐Wackernagel, 2006), and pigeons that fly far from their nesting site one day may move very little the next day (Johnston & Janiga, 1995). Cities with higher human population density tend to produce more food waste for pigeons to feed on, while pigeons in less densely populated cities may need to cover more distance or fly outside the city to acquire the same nutritional value as pigeons in more densely human populated areas. Moreover, pigeons may frequent locations where deliberate supplemental feeding (i.e., the intentional placement of food) occurs consistently. Juveniles learn about feeding locations from their parents and may become separated from their parents during these initial feeding flights and fail to return home, thus resulting in a natal dispersal distances longer than what young pigeons would attempt on their own. This phenomenon is poorly documented, and the studies that have been conducted reported short natal dispersal distances of less than 100 meters (reviewed in Johnston & Janiga, 1995).

Landscape features have also been shown to contribute to pigeon density and distribution within an urban environment. Multiple studies have found the highest density of pigeons in urban cores, where human population density and percent impervious surface are the highest (Hetmański, Bocheński, Tryjanowski, & Skórka, 2011; Przybylska et al., 2012; Sacchi, Gentilli, Razzetti, & Barbieri, 2002). It is possible that pigeons within urban cores are less likely to disperse because of this concentration of resources. Alternatively, high resource availability that supports large pigeon populations in urban cores could lead to intense resource competition and subsequent dispersal. Presently, it is unclear what spatial genetic pattern will emerge in a nearly continuous urban area, with multiple urban cores and varying resource availability.

Previous studies on feral pigeon population genetics have used uniform sampling within a single city (Tang et al., 2018) and lumped together a priori groups of pigeons from multiple cities (Jacob et al., 2015) to understand the spatial genetic patterns of pigeons. While these methods provide insight into the processes that govern observed patterns, different patterns may emerge from studying pigeons across multiple neighboring cities. Here, we sample pigeons from urban cores across the Northeastern megacity and use reduced representation genome sequencing (i.e., ddRAD) to answer the following specific questions: (1) Are pigeons comprised of multiple, separately evolving populations in the Northeastern megacity or a single population? (2) Do pigeons in the Northeastern megacity exhibit panmixia, isolation by distance, or isolation by barrier? (3) If there is genetic structure among pigeons within the Northeastern megacity, what landscape factors are potentially contributing to this structure?

## 2 METHODS

### 2.1 Sample collection

**FIGURE 1:**
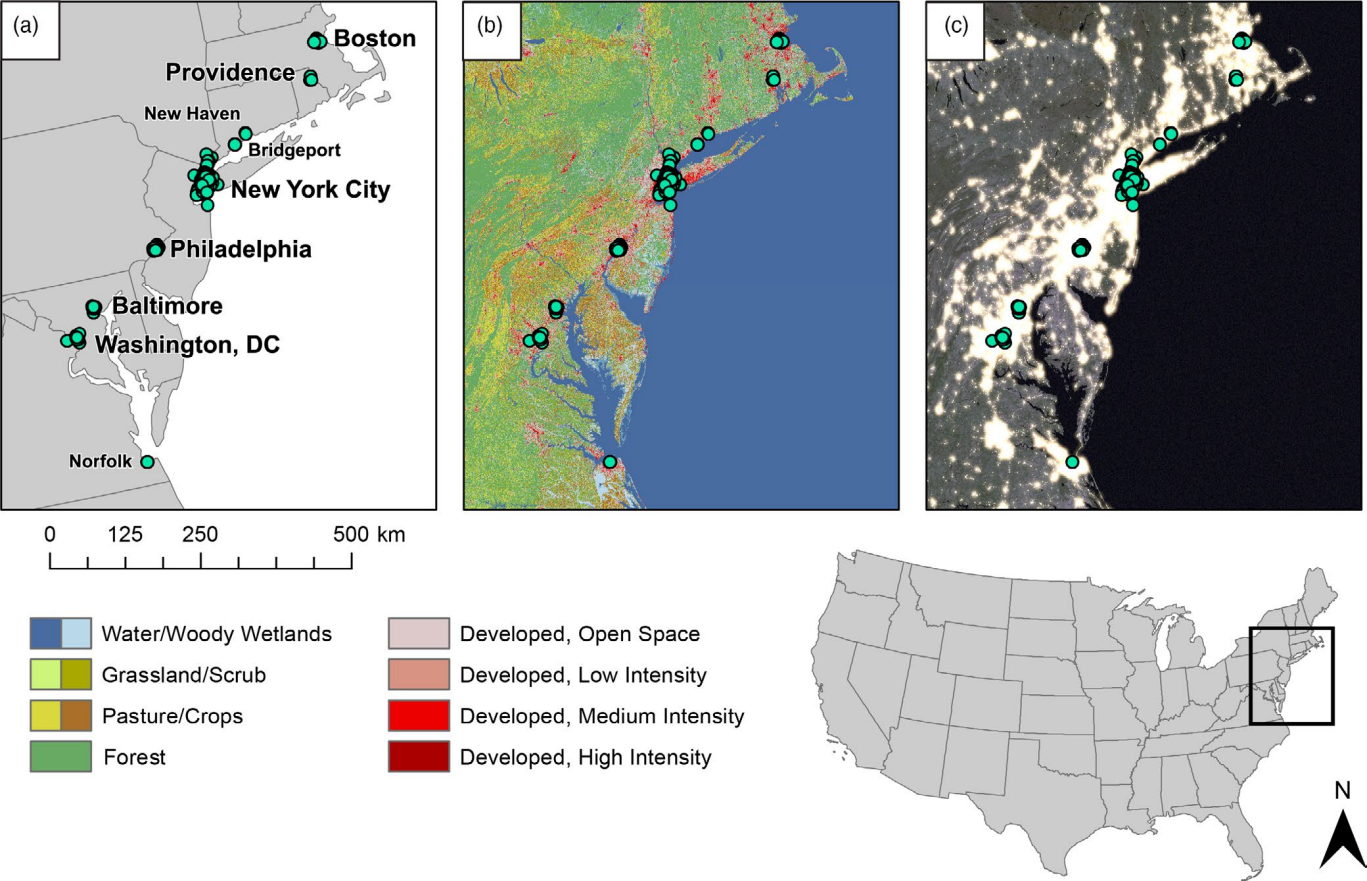
Maps showing sampling location including (a) cities where samples were collected, (b) National Land Cover Data (NLCD v.2011; Homer et al., 2015), and (c) light intensity at night (NASA Earth Observatory, 2017). Sample locations are shown as teal circles. Note that while National Land Cover Data (NLCD) is often used to indicate urbanization, NASA’s Earth Observatory images of artificial light at night shows the interconnectedness of urbanization that is missed by landcover data. A more colorblind friendly version of this figure appears in the supplement (Figure S1)

We focused our sampling on six metropolitan areas within the Northeastern megacity (listed from north to south): Boston, MA; Providence, RI; New York City, NY; Philadelphia, PA; Baltimore, MD; and Washington, DC (Figure 1). We decided to focus on these regions since previous research showed that pigeons occurred in the highest densities in areas with a large number of humans and high‐rise buildings over four floors (Przybylska et al., 2012; Tang et al., 2018). We sampled in three additional smaller cities: Norfolk, VA; Bridgeport, CT; and New Haven, CT, for a total of nine metropolitan areas. Two of these smaller cities (Bridgeport, CT; and New Haven, CT) are located in between major metropolitan areas, while the third (Norfolk, VA) is located at the southern end of the region, allowing us to capture diversity between and beyond the larger metropolitan areas. To collect pigeons, we drove or walked around each city using the smartphone application MapMyWalk (Under Armour, Inc., Baltimore, MD) to track areas we had covered. When we spotted pigeons, we used commercially available bird seed to attract multiple individuals to congregate on the ground and then used a net gun (TheNetGunStore.com, Broken Arrow, OK) to capture pigeons alive. The net gun propels a weighted net over the target, capturing the birds for further analysis. We used a 21‐gauge needle to draw blood from the ulnar vein following techniques outlined in Gaunt and Oring (1997) and Owen (2011). We stored blood in RNA later and placed it in a −20°C freezer until DNA extraction. To reduce the chance of resampling the same individual, we banded each pigeon with an aluminum band (National Band & Tag Company, Newport, KY) that contained a unique ID. In addition to our own sampling, we obtained blood samples from a wildlife rehabilitation center in New York City and tissue samples from the United State Department of Agriculture (USDA) Animal and Plant Health Inspection Service (APHIS) program at airports in New York City, New York and Norfolk, Virginia. Tissue samples (either muscle or toe) were stored in 99% EtOH and placed in a −20°C freezer until DNA extraction. All animal handling procedures were approved by the Institutional Animal Care and Use Committee at Fordham University (Protocol No. #EJC‐17–01) and local agencies where applicable (Connecticut Department of Energy & Environmental Protection permit #1718009; Maryland Department of Natural Resources permit #56952; New York State Department of Environmental Conservation permit #2003; and Rhode Island Department of Environmental Management permits #2018‐04‐W and #2019‐02‐W).

### 2.2 ddRAD‐seq library preparation

We extracted DNA from blood and tissue samples using the DNeasy Blood and Tissue Kit (Qiagen, Germantown, MD, USA) according to the manufacturers protocol with the following modifications: (a) When using blood, we used 50 µl of blood, (b) when using tissue, we let tissue samples incubate with Proteinase K overnight, (c) we added 4 µl of RNase A to each extraction after incubation, (d) we heated the AE buffer to 75°C before placing it on the filter, and (e) we let the AE buffer sit on the filter for approximately 1 hr before eluting. We performed two elutions of 100 µl each that we then combined. We used an Infinite 200 Pro NanoQuant (Tecan Group Ltd., Männedorf, Switzerland) to quantify the amount of DNA present in each sample. We then digested each sample with the restriction enzymes SphI and MluCI and prepared ddRAD libraries following a protocol adapted from Peterson, Weber, Kay, Fisher, and Hoekstra (2012) and selecting for fragments between 376 bp–412 bp using a Pippin Prep (Sage Science, Beverly, MA). In total, we digested 473 samples that were combined into nine pools of 48 samples each and one pool of 41 samples. These pools were then sequenced on two lanes of a HiSeq 4,000 (Illumina, Inc., San Diego, CA, USA) at the Translational Genomics Research Institute (Phoenix, AZ, USA) using paired‐end 125 bp sequencing.

### 2.3 Data processing and SNP calling

We used STACKS v2.3d (Rochette, Rivera‐Colón, & Catchen, 2019) to sort, filter, and demultiplex reads with the *process_radtags* script using the individual barcodes that were ligated during the ddRAD library preparation. We aligned reads to the most recent pigeon reference genome, Cliv_2.1 (Holt et al., 2018) using BOWTIE2 v2.3.4.3 (Langmead & Salzberg, 2012) with the default parameters. We used SAMTOOLS (Li et al., 2009) to convert files from .sam to sorted .bam files. Reads for each individual aligned at a rate greater than 50%; therefore, all individuals were retained for downstream analyses (mean = 86.70%, *SD* = 0.036). We built the initial catalog using all reference aligned samples with the *ref_map.pl* pipeline in STACKS (per‐sample coverage mean = 7.8×, *SD* = 2.4×). Next, we created our SNP dataset using the *populations* script with the following filters for retaining SNPs: (a) Only loci genotyped in at least 8 out of 9 metropolitan areas (to limit missing data), (b) only loci found in 75% of samples (to limit missing data), (c) only SNPs with minor allele frequency greater than or equal to 5% (to ensure that rare SNPs, likely due to errors in SNP calling, are excluded), and (d) only one SNP per locus (to avoid extreme linkage between SNPs) (Rochette & Catchen, 2017). Following this filtering, we retained 35,200 SNPs for downstream analyses.

We then used PLINK v1.9 (Chang et al., 2015) to calculate pairs of individuals that were related at greater than 50% identity, thereby identifying sibling and parent–offspring relationships. We removed these individuals and ran our preliminary analysis on both the whole dataset and the dataset with one individual from each of the sibling/parent–offspring pairs removed. We found no differences in our preliminary analysis between these two datasets; therefore, we proceeded with the entire dataset for final downstream analyses.

### 2.4 Data analysis

#### 2.4.1 Genetic diversity and effective population size

We also used the *populations* script in STACKS to calculate summary statistics for the entire dataset of 473 individuals. Using the ‐‐fstats flag in STACKS, we calculated indices of genetic diversity for the entire Northeast population, including fixation index (*F*_ST_), expected heterozygosity (*H_E_*), observed heterozygosity (*H_O_*), nucleotide diversity (π), and inbreeding coefficient (*F*_IS_). Additionally, we used the linkage disequilibrium method implemented in NEESTIMATOR v2.1 (Do et al., 2014) to estimate effective population size (*N_e_*). To reduce computing time, we randomly selected five subsets of 10,000 SNPs for our NEESTIMATOR analysis.

#### 2.4.2 Genetic structure across the northeastern megacity

To investigate the diversity between groups of individuals, we ran a discriminant analysis of principal components (DAPC) using the R package *adegenet* (Jombart, 2008; Jombart & Ahmed, 2011). DAPC is a multivariate approach that calculates principal components and summarizes the difference between evolutionary clusters while minimizing variation within a cluster (Jombart, Devillard, & Balloux, 2010). This approach maximizes the diversity between groups of individuals in order to visualize the between group differences. DAPC does not rely on population genetic models and, therefore, is free of assumptions about Hardy–Weinberg equilibrium and linkage disequilibrium. To compute our DAPC, we first identified the number of genetic clusters by transforming the data using PCA. We explored our data specifying that we wanted to evaluate up to 40 clusters, since this number is well beyond the number of clusters we expected to find in our data. *adegenet* runs a k‐means algorithm with increasing values of k and computes BIC. Based on the lowest BIC value, we selected *k* = 2 clusters for our final analysis. Next, we performed a DAPC using these two clusters. When there is a small number of clusters, all eigenvalues can be retained for discriminant analysis; therefore, we retained all eight discriminant functions. We selected 21 as our optimal number of retained principal components and then recalculated the DAPC using 21 principal components and eight discriminant functions. We then repeated this analysis with 70 random samples from NYC and samples from all other cities.

We used ADMIXTURE v1.3.0 (Alexander, Novembre, & Lange, 2009), which uses a likelihood model approach to estimate ancestry, to describe the population structure in our sample. We ran ADMIXTURE using the ‐‐cv flag to enable cross‐validation and examine the entire dataset for *K* = 1 to *K* = 15, running each *K* value for five iterations. The lowest values for cross‐validation error indicate the most likely values of *K* (Alexander & Lange, 2011), although this approach may not always detect the single best value of *K* (Lawson, van Dorp, & Falush, 2018). We then used the R package *ggplot* to visualize stacked bar plots for all K values. To examine how oversampling New York City may have influenced our, we also thinned our New York City sample to 70 random individuals and reran ADMIXTURE with all samples from other cities and the 70 samples from New York City.

#### 2.4.3 Movement and dispersal in the Northeastern megacity

To investigate isolation by distance, we used the R package *adegenet* to conduct a Mantel test (Jombart, 2008). A Mantel test examines the correlation between pairwise matrices (Mantel, 1967), in this case, pairwise genetic distance and geographic distance. This analysis allowed us to assess the possibility of isolation by distance between pigeons in geographically distant cities. We used permutation testing (10,000 permutations) to check for significance of the Mantel test. We also examined our data using a Mantel correlogram visualized with the *ecodist* package in R (Goslee & Urban, 2007). A correlogram is a spatial autocorrelation method that examines the relationship among variables (allele frequencies) at different geographic distance classes or “steps.” Since we did not have an a priori assumption of step sizes, we examined the following step sizes: 100 m, 500 m, 1 km, 5 km, 10 km, 15 km, 20 km, 25 km, and 50 km. To test whether our patterns were driven by closely related individuals, we repeated this analysis with individuals that were over 50% related removed from the dataset.

To visualize deviations from isolation by distance among pigeons across our study area, we used estimated effective migration surfaces (EEMS) (Petkova, Novembre, & Stephens, 2016). EEMS represents genetic differentiation as a function of migration rates and produces visualizations of geographic regions that deviate from isolation by distance. More specifically, this approach assumes isolation by distance as the null model and deviations from the null represent high effective migration (i.e., possible corridors for gene flow) or low effective migration (i.e., possible barriers to gene flow). To execute EEMS, we first used PLINK to create a .bed file from the .ped and .map files generated by *populations* in STACKS. We then used *bed2diffs* to create a dissimilarity matrix. Because the dissimilarity matrix used by EEMS requires that no genotypes are missing, we multiplied the observed allele frequency at a particular SNP by two for any individual that was missing data at a particular locus. We created an outer coordinate file in ArcMap to define the range that we sampled that approximately followed the shoreline of the Northeastern United States from Norfolk, VA to Boston, MA, including areas of water between mainland areas that pigeons are able to transverse (e.g., Chesapeake Bay, Delaware Bay, and the Long Island Sound). The EEMS documentation recommends running the model multiple times, varying the number of demes; therefore, we examined deme sizes of 200, 300, 400, 500, 600, 700, 800, 900, and 1,000. For each deme size, we first optimized the proposal variances by tweaking parameters so that proposals were accepted about 20% to 30% of the time and the MCMC chain converged. Once optimized, we then repeated the analysis for each deme two more times, using a random seed each time for a total of 28 runs. We used an MCMC length of 12,000,000 iterations with a burn‐in of 4,000,000. We then visualized the convergence of runs and merged all 28 runs into a single plot using the R package *reemsplots2* and produced maps of effective migration rate (*m*) and effective diversity (*q*).

## 3 RESULTS

### 3.1 Genetic diversity and effective population size

Across the Northeastern megacity, pairwise *F*_ST_ values were low (0.002–0.047) indicating weak population genetic differentiation (Table S1). Across all sampled cities, pigeons had a positive but low inbreeding coefficient (*F*_IS_) ranging from 0.010 to 0.041. We estimated values of observed heterozygosity (*H_o_*), expected heterozygosity (*H_e_*), and nucleotide diversity (π) for the nine cities that were sampled and found slight regional differences (Table S2). We found mean estimated effective population size ranged from 2,500.8 in New York City to 44.42 in Bridgeport (Table S3) though sample size may be driving these results. We were unable to calculate effective population sizes in New Haven and Norfolk due to low sample sizes in each of these cities.

### 3.2 Genetic structure across the Northeastern megacity

**FIGURE 2:**
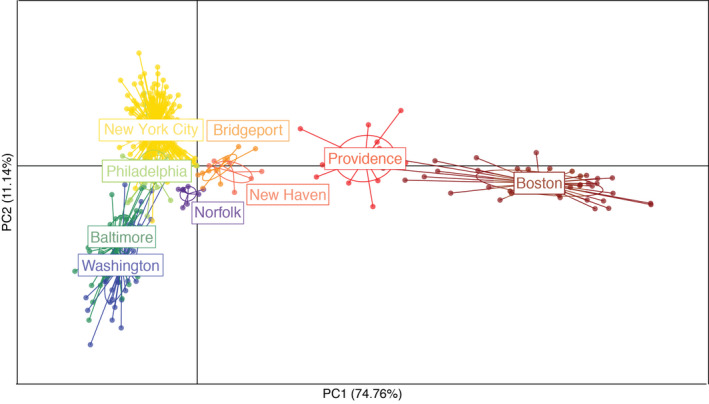
Discriminant analysis of principal components (DAPC) for 35,200 genome‐wide SNPs recovered from pigeons. This graph recapitulates geography, showing separation of sampled collected in the northern part of the megacity (Boston and Providence) from samples collected in more southern parts of the megacity along the 1st PC axis (*x*‐axis) and New York City/Philadelphia samples separating from the Baltimore/Washington DC samples along the 2nd PC axis (*y*‐axis)

DAPC identified two clusters. The first cluster (*n* = 60) contained all the Boston samples and 12 of the Providence samples; the second cluster (*n* = 413) contained all other samples. Visualization of the DAPC as a scatter plot (Figure 2) shows differentiation along the first principal component axis (horizontal axis) separating samples collected in Boston and Providence from samples collected in more southern cities. Differentiation along the second principal component axis (vertical axis) separates Norfolk, Baltimore, and Washington, DC, from Philadelphia and New York City. Together, these two axes explain 85.9% of the variation found in our dataset. Our thinned dataset showed similar results (Figure S2).

**FIGURE 3:**
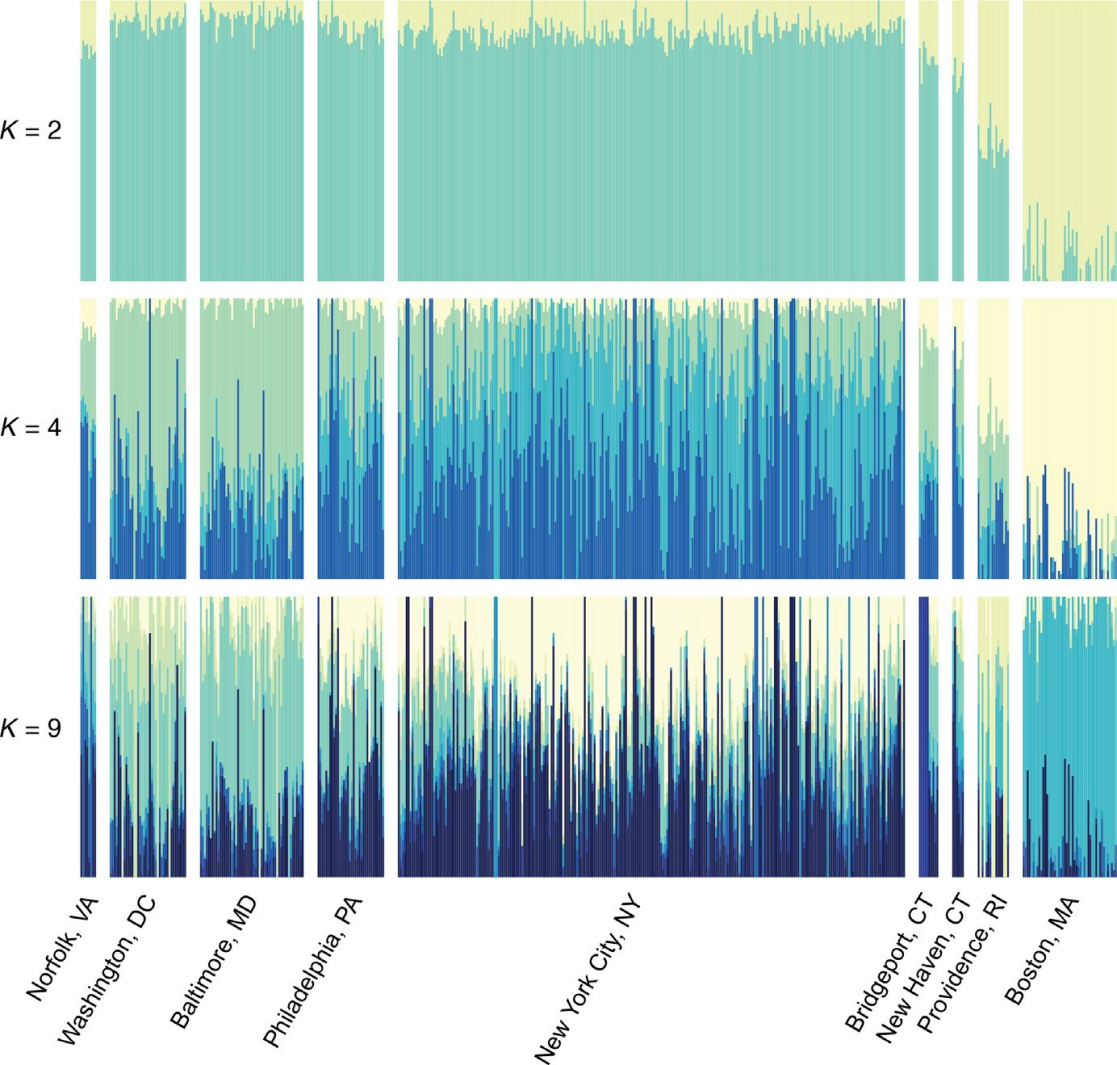
Bar plots showing our ADMIXTURE v1.3.0 (Alexander et al., 2009) results at *K* = 2, 4, and 9. At *K* = 2, samples from the northern region of the Northeastern megacity (Boston) show a different shared ancestry from samples collected in more southern regions of the megacity. At *K* = 4 and *K* = 9, samples from Boston continue to show a different shared ancestry than samples from the rest of the megacity. Cross‐validation indicated that *K* = 2 is the most well‐supported K value based on analysis of 35,200 SNPs genome‐wide SNPs

Our cross‐validation error analysis in ADMIXTURE indicated that *K* = 2 was the most well‐supported *K* value. Our ADMIXTURE plot (Figure 3) at *K* = 2 shows differentiation between Boston and all other cities, with some admixture in Providence. Our ADMIXTURE plot*K* = 4 also shows differentiation between Boston samples and samples from more southern cities; however, with these higher *K* values there is increased admixture in cities south of Boston, indicating high levels of genetic connectivity between the majority of the cities that were sampled (Figure S3). When we thinned our samples from New York City to 70 samples to test how oversampling may have influenced our ADMIXTURE results, our cross‐validation analysis once again indicated that *K* = 2 was our most well‐supported K value and out ADMIXTURE plot with this dataset recapitulates the differentiation that we found in our full dataset (Figure S4).

Taken together, the results of the DAPC and ADMIXTURE show a separation between Boston samples and samples from more southern cities. Providence samples have ancestry that is mixed between Boston and southern cities. We found there is a high degree of admixture between pigeons in Norfolk, Washington, DC, Baltimore, Philadelphia, New York City, Bridgeport, and New Haven indicating gene flow between these regions.

**FIGURE 4:**
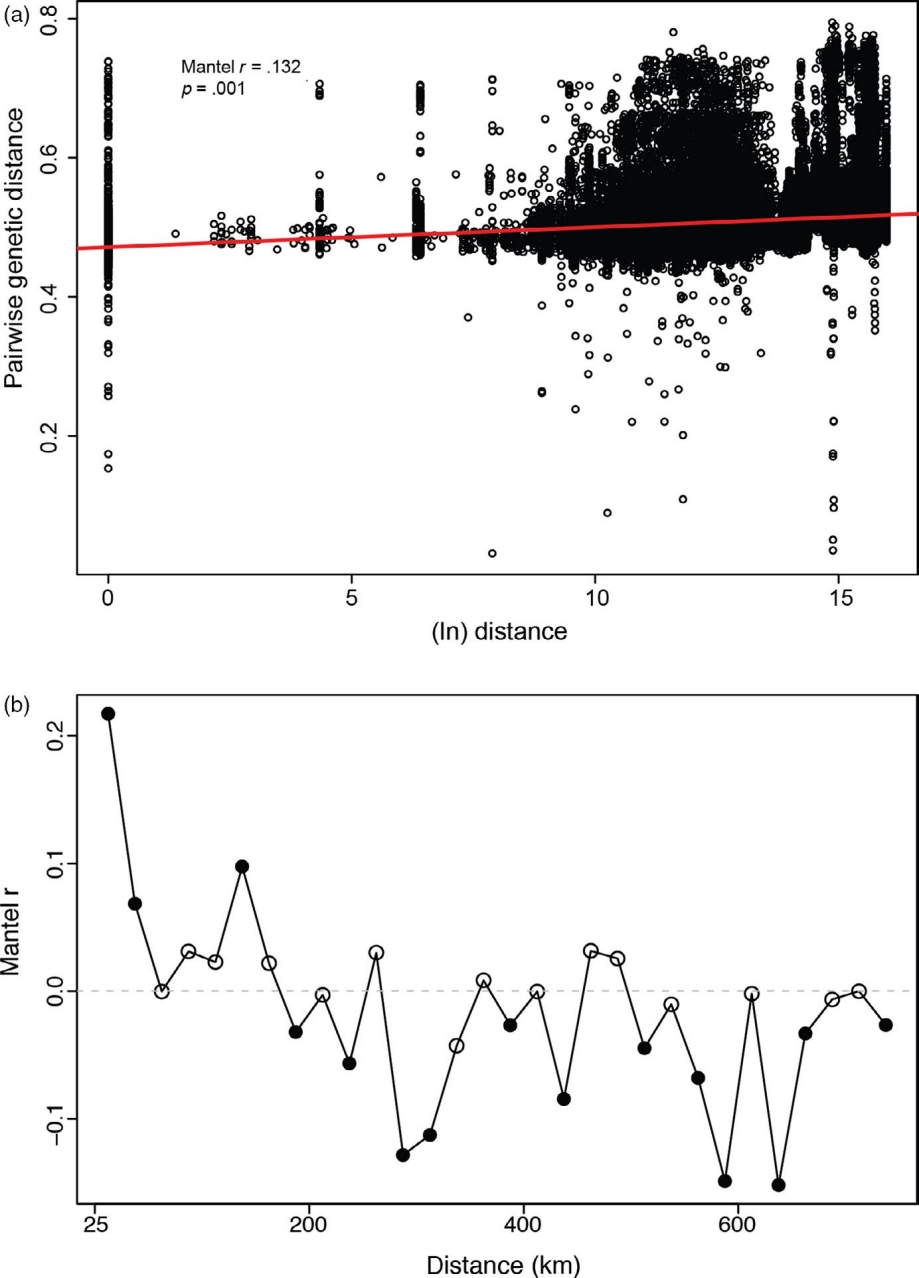
(a) Scatterplot of the natural log of Euclidean geographic distance between samples and pairwise genetic distance showing weak isolation by distance relationship. The trend line (shown in red) depicts the linear relationship calculated by a simple Mantel test (Mantel *r* = 0.132, *p* = .001). (b) Mantel correlogram showing the relationship between genetic distance and geographic distance within each 25 km distance class. Each circle represents a distance class, and filled circles represent significant associations (α = 0.05)

A Mantel test showed a weak isolation by distance relationship and positive spatial autocorrelation (Mantel *r* = 0.132, *p* = .001) (Figure 4a). A Mantel correlogram showed decreasing genetic relatedness over geographic distance (Figure 4b). From 0–50 km, we found positive and significant spatial autocorrelation, with extremely high correlation between pairs within 0–25 km (*r* = 0.22, *p* = .001). We found that after 50 km, pigeons are no more related than they would be at random. When we repeated this analysis with related individuals removed, we found a similar pattern (pairs within 0–25 km *r* = 0.23, *p* = .001).

**FIGURE 5:**
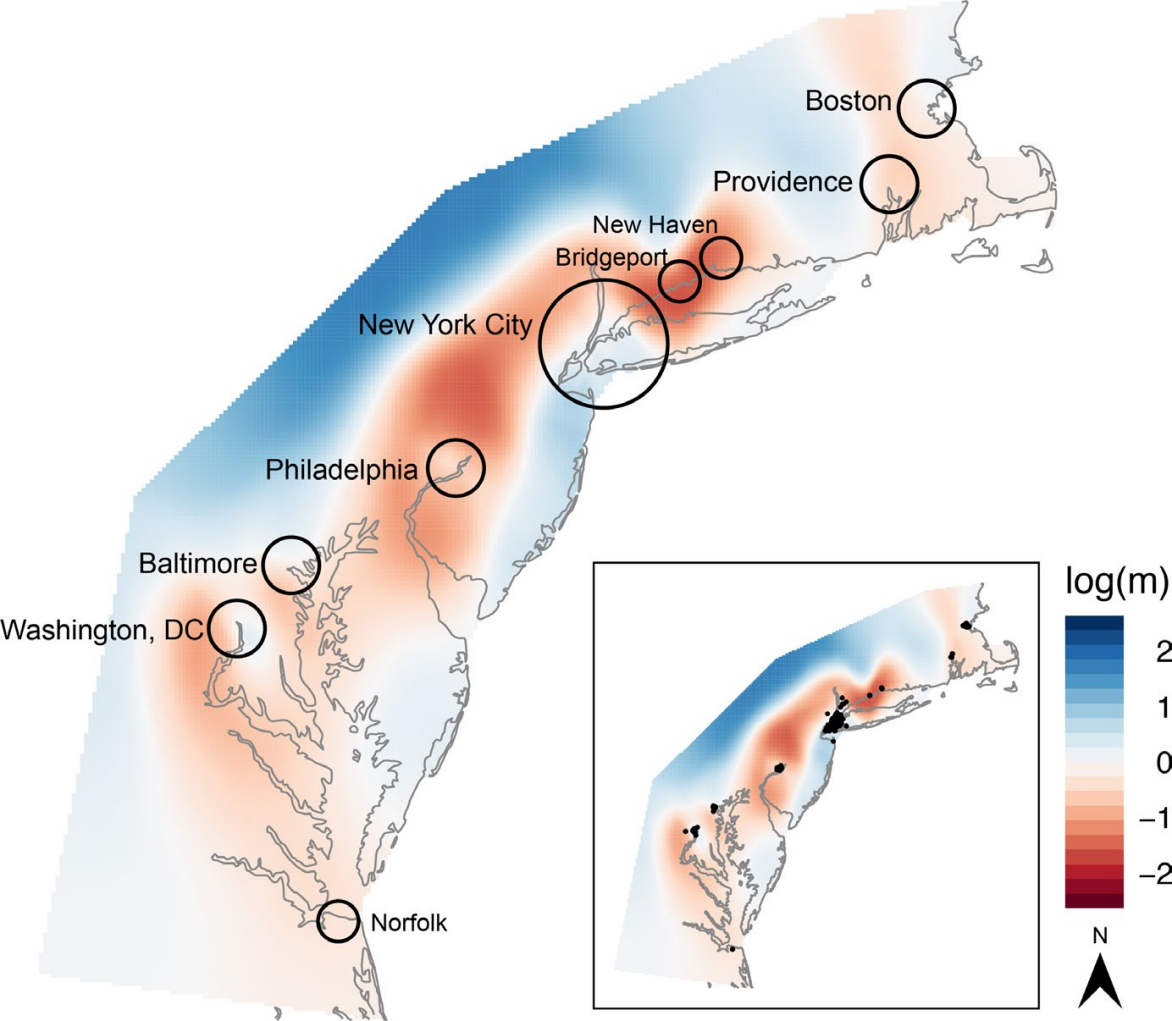
Estimated effective migration surface (EEMS) for pigeons in the Northeastern United States. Coloring of the map represents relative effective migration rates ranging from higher‐than‐average (blue) to lower‐than‐average (red) historic gene flow with isolation by distance represented as the null (white). Circles represent the approximate sampling range for each city. Within many cities, there is high‐than‐average gene flow and isolation by distance, but there is lower‐than‐average gene flow between cities. While this map shows higher‐than‐average gene flow to the west, we are unable to draw conclusions from this area since it was not sampled. Inset map shows sample locations in black

We used EEMS to visualize deviations from isolation by distance. Unlike PCA, EEMS takes into account sampling locations when modeling regions of higher‐than‐average and lower‐than‐average historic gene flow. EEMS uses isolation by distance as a null model and represents deviations from isolation by distance in either blue (higher‐than‐average gene flow) or red (lower‐than‐average gene flow). Our EEMS analysis shows high‐than‐average gene flow and isolation by distance within most of the cities we sampled, but lower‐than‐average gene flow between cities (Figure 5).

## 4 DISCUSSION

In this study, the use of ddRAD sequencing allowed us to examine the movement of pigeons and the evolutionary processes that shape feral pigeons at a much higher resolution than has previously been attempted for any urban region. We found a moderate clinical pattern in pigeons along a north–south axis. Feral pigeons in the Northeastern megacity form two genetic clusters, with pigeons from Boston, MA, showing genetic differentiation from pigeons in more southern cities. In Providence, RI we found evidence of admixture between Boston pigeons and pigeons from further south, indicating that there are unlikely to be any complete barriers to gene flow between Boston and cities further south. Overall, feral pigeons in the Northeastern megacity maintain high genetic connectivity over a large urbanized region, likely due to their ability to move through human‐dominated landscapes.

Our results must be interpreted in relation to our sampling scheme. We were unable to sample pigeons in many of the smaller municipalities between major cities, which may have influenced our Mantel correlogram and EEMS results. However, many of these smaller cities and towns had few to no pigeons, thus making it difficult to conclude whether the patterns we found were due to inadequate sampling effort or simply few pigeons existing outside major cities. Even within the larger cities that we sampled, we found pigeons were concentrated in the downtown areas where human population density and activity was at its highest.

### 4.1 Genetic diversity and effective population size

Organisms with limited dispersal abilities (e.g., most amphibians, small mammals) are expected to have low genetic diversity in areas where habitat fragmentation caused by urbanization prevents organisms from successfully dispersing and reproducing (Miles et al., 2019; Munshi‐South, Zak, & Pehek, 2013; Wilson, Farley, McDonough, Talbot, & Barboza, 2015). In contrast, urban organisms that can disperse through a broad range of habitat types, such as large mammals and birds, are less likely to experience severe declines in genetic diversity (Blanchong, Sorin, & Scribner, 2013; Unfried, Hauser, & Marzluff, 2013). Despite previous research suggesting that pigeons do not have large daily movements (Rose et al., 2006; Sol & Senar, 1995), we found pigeons in the Northeastern United States have high genetic diversity that likely reflects substantial dispersal distances by some individuals across the urban landscape. We also found no private alleles and low pairwise *F*_ST_ values, indicating little genetic differentiation between cities. Miles et al. (2018) also found high levels of genetic diversity in urban western black widow spiders (*Latrodectushesperus*) likely due to human transportation networks that facilitate dispersal within cities. Similarly, Combs, Byers, et al. (2018) found that brown rats (*Rattus norvegicus*) in four cities (Salvador, Brail; New Orleans, USA; New York City, USA; and Vancouver, Canada) exhibited high levels of genetic diversity within each city, likely attributed to connectivity and large effective population sizes within the urban habitat. Additionally, brown rats exhibited some evidence of occasional long‐distance dispersal, despite short (200 m) dispersal distances being more common (Combs, Byers, et al., 2018; Combs, Puckett, et al., 2018).

Effective population size (*N_e_*) is helpful for calculating the rate of evolutionary change in a population caused by genetic drift. *N_e_* is used to determine genetic variability within a population and the effectiveness of selection relative to genetic drift (Charlesworth, 2009). However, N_e_ is notoriously difficult to calculate and does not necessarily represent the actual census population size (Charlesworth, 2009; Frankham, 1995; Schwartz, Tallmon, & Luikart, 1998). Moreover, low sample sizes, though not oversampling, may influence *N_e_* estimates (Marandel et al., 2020). Nevertheless, we found that N_e_ was highest in New York City and lowest in Bridgeport, Connecticut. The low effective population size in Bridgeport corresponds with the break in genetic clusters that we see in our data, with the more northern cities of Boston and Providence clustering together and separate from the more southern cities (Figure 3). Likewise, Combs, Puckett, et al. (2018) found local differences in *N_e_* for brown rats in Manhattan, with a low effective population size in Midtown, which correlated with a break in the population that distinguished the uptown genetic cluster from the downtown genetic cluster.

### 4.2 Genetic structure across the northeastern megacity

Organisms that rely on humans, such as German cockroaches (*Blattellagermanica*), bed bugs (*Cimex lectularius*), and brown rats, likely reflect human dispersal and settlements in their own patterns of dispersal and population genetic structuring. However, the connectivity between populations of these commensal organisms will depend on their ability to disperse alongside humans and/or though natural habitat. For example, German cockroaches are acutely adapted for indoor habitats, but are not known to exist as self‐sustaining populations in the natural environment anywhere within its considerable range (Roth, 1985). Previous studies have demonstrated that cockroaches are more likely to disperse within a building than between buildings (Crissman et al., 2010). Similarly, bed bug infestation patterns show that bed bugs can actively disperse within a building by crawling between rooms or passively across larger distances via human‐mediated transport (i.e., in luggage or used furniture) (Booth et al., 2012; Saenz, Booth, Schal, & Vargo, 2012). Moreover, brown rats are able to navigate both natural terrestrial environments (e.g., parks) and fabricated anthropogenic environments (e.g., subways, buildings) leading to patterns of isolation by distance. However, because of their ability to fly pigeons are able to more easily maneuver through the city landscape.

Due to their ability to easily transverse the anthropogenic environment, pigeons within the city of Paris and the city‐state of Singapore have been shown to comprise a single population within each city (Jacob et al., 2015; Tang et al., 2018). We found there is very little genetic differentiation across our entire study area. This is somewhat surprising given that the Northeastern United States is approximately 200 times larger than Singapore, and pigeons have been in North America for approximately four times longer than pigeons in Singapore. We suspect that the genetic pattern we observed is due to the intensity of urbanization across the Northeastern landscape. Specifically, the Northeastern United States consists of large cities connected by suburban areas that may provide nearly continuous habitat for pigeons across hundreds of kilometers. Hofmeister et al. (2019) found that European starlings (*Sturnus vulgaris*), another common urban species, showed low genetic structure across 17 populations spanning the entire United States, suggesting that the patterns observed in starlings and pigeons may be common across non‐native birds.

Our multivariate clustering (DAPC) and maximum likelihood estimation of individual ancestries (ADMIXTURE) both detected two genetic clusters—one cluster containing the samples from Boston and Providence, and a second cluster containing samples from more southern cities. Our study found a divide in pigeons that recapitulates the break in urbanization found between New Haven, Connecticut, and Providence, Rhode Island (Figure 1). This area is more forested and has less high intensity development indicating that considerable urbanization may be necessary to maintain gene flow in pigeons across cities. These two clusters may also represent statistical artifacts of the clinical nature of our sampling scheme, with Boston and Providence samples separating from New York City and Mid‐Atlantic samples due to isolation by distance. However, due to the difficulty finding and catching pigeons in less urban areas, we suspect that landscape features, in part, contribute to the population structuring of pigeons.

The previous multicity study, conducted across Spain, France, and Switzerland, found that dispersal between cities was rare and that individuals living in geographically distant cities were unlikely to be related (Jacob et al., 2015). Our results likely differ because agricultural subsidies in Europe have led to European cities being far less connected by urban sprawl than cities in the Northeastern United States (Lewyn, 2009; Richardson & Bae, 2016). The Northeastern United States is highly urbanized with the area from Boston, MA to Washington, DC containing 17% of the United States population, across only two percent of the United States landmass (Regional Plan Association, 2006). Since only one migrant per generation is theoretically needed to maintain panmixia, minimal movement between municipalities may lead to the patterns observed in our dataset.

Previous research on the impacts of urbanization on avian populations has found urbanization often leads to population fragmentation (Delaney, Riley, & Fisher, 2010; Fernández‐Juricic, 2004; MacDougall‐Shackleton, Clinchy, Zanette, & Neff, 2011; Sadanandan & Rheindt, 2015). This result is surprising given the ability of birds to disperse as juveniles and adults through or around urbanization. However, a handful of studies have shown that urbanization may not limit dispersal in all birds (Björklund, Ruiz, & Senar, 2010; Partecke, Gwinner, & Bensch, 2006; Zhang, Suo, Liu, & Liang, 2013); thus, the impact of urbanization may be dependent on the specific life history strategies of an organism (Miles et al., 2019). Research on juvenile and adult dispersal in pigeons is relatively limited. A banding study of pigeons in Lawrence, Kansas, estimated that the mean natal dispersal distance of pigeons is 93 m, though the authors mention that this is likely an underestimate (Johnston & Janiga, 1995). Pigeons have been observed traveling up to 25 km from the city to surrounding fields to feed, though most studies of marked individuals have found that pigeons travel relatively short distances, with one study recording a maximum distance of only 0.34 km (reviewed in Rose et al., 2006). Our study found that pigeons within 25 km are likely to be highly related, but outside this range relatedness decreases rapidly and is no longer significant at 75 km. Captive homing pigeons have been recorded flying over 1,500 km (Walcott, 1996) though such movement is unlikely to occur in feral pigeons which likely lack the morphology and need to move such large distances (Johnston & Janiga, 1995). We did find some stochasticity in our Mantel correlogram, which may be due to clumped sampling, since we were unable to find and catch pigeons in portions of our study range. Feral pigeons tend to occur in high density near urban cores (Sacchi et al., 2002) where food and nesting resources are in high abundance (Tang et al., 2018). Since urban cores are not evenly distributed throughout the Northeastern United States, we suspect this unevenness could be creating this pattern.

Our EEMS analysis detected lower‐than‐expected gene flow between cities and higher‐than‐expected gene flow within cities, though we can only draw conclusions between sampled areas and not from areas that were unsampled. This pattern is consistent with our Mantel correlogram which showed that pigeons are often related at a distance up to 50 km. This finding is also consistent with previous research on feral pigeons in Europe which found that pigeons within cities are highly related and do not show genetic differentiation (Jacob et al., 2015). Our EEMS analysis showed that larger cities, which tended to have higher sample sizes, often had higher‐than‐expected gene flow within the city. While this finding may be a result of sampling bias, this pattern could also result from intraspecific resource competition. Larger cities tend to have more resources available, allowing the pigeon flocks to increase in both the number of flocks and the number of birds within flocks. This increase then leads to resource competition, forcing some members of the flock to move to other areas. Previous research found that high density of breeding pairs within a natal colony led young pigeons to disperse (Hetmański, 2007). Young pigeons that moved from a colony exhibited much higher reproductive success than individuals that remained in their natal colony. Hetmański (2007) points out that dispersal costs are relatively low in this case, since pigeons encounter few diurnal predators and new nesting sites are often plentiful in urban environments.

Similarly, Hofmeister et al. (2019) found low migration rates among European Starlings in the United States, with higher‐than‐expected migration found mostly in areas where the researchers had not sampled starlings. European Starlings are extremely common in urban and suburban areas (Fischl & Caccamise, 1985), and their preference for anthropogenic habitat may explain the observed pattern of low migration between cities. Brown rats are another common human commensal that relies on humans throughout most of their range. Combs, Puckett, et al. (2018) used EEMS to explore gene flow among brown rats living on the island of Manhattan in New York City and found reduced areas of gene flow in Midtown, where human residential density is lower.

Taken together, these studies and results from pigeons suggest that organisms dependent on humans exhibit fine‐scale spatial genetic patterns that reflect human patterns and distribution. Thus, the implications of urbanization on wildlife are dependent upon the life history traits of the organism, how humans are distributed throughout the landscape, and the extent of urbanization (Bonier, Martin, & Wingfield, 2007; McKinney, 2006). Moreover, while urbanization is often thought of in regard to habitat fragmentation, the movement of some organisms is facilitated by urbanization (Miles et al., 2019). Our study indicates that pigeons are moving between municipalities more than previously observed, and in the Northeastern United States, this movement may be facilitated by extensive urbanization. This study is the first to our knowledge that demonstrates how urbanization across the Northeastern megacity facilitates gene flow in a human commensal.

## Supporting information

Appendix S1

## Data Availability

Data for this study are available at National Center for Biotechnology Information (NCBI) Sequence Read Archive (SRA), accession PRJNA607495.
