# Supplementary material for: Widespread genetic connectivity of feral pigeons across the Northeastern megacity
Source: Evol Appl. 2020 Apr 23;14(1):150–62. doi: 10.1111/eva.12972 (PMC7819573; doi:10.1111/eva.12972)
Supplement: Supplementary file 1 — Appendix S1 [file EVA-14-150-s001.docx]

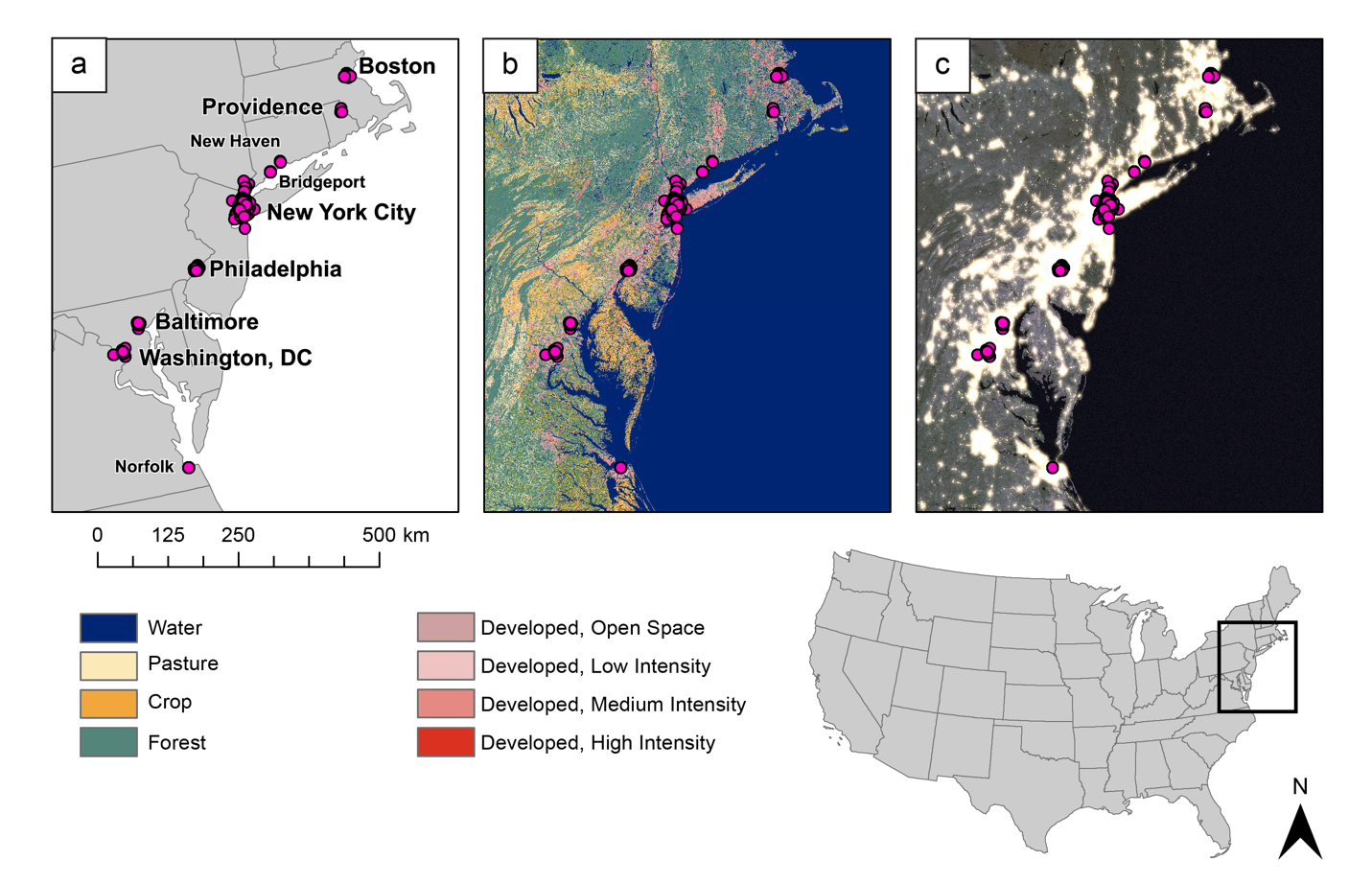


Figure S1: Colorblind friendly version of Figure 1 showing sampling location including (a) cities where samples were collected (b) National Landcover Data (NLCD v.2011; Homer et al., 2015) and (c) light intensity at night (NASA Earth Observatory, 2017). Sample locations are shown as teal circles. Note that while national landcover data (NLCD) is often used to indicate urbanization, NASA’s Earth Observatory images of artificial light at night shows the interconnectedness of urbanization that is missed by landcover data.


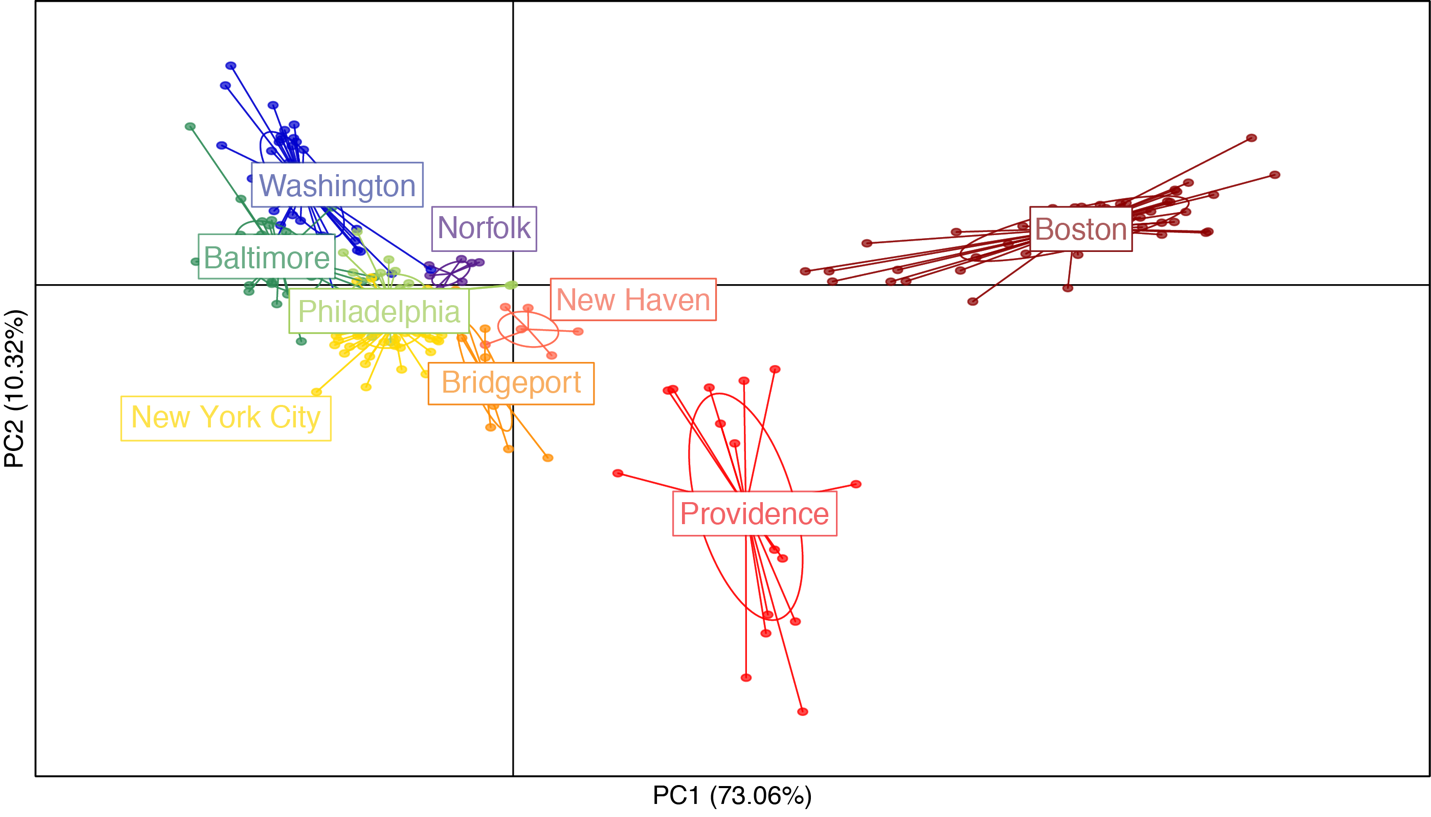


Figure S2: Discriminant analysis of principal components (DAPC) SNPs recovered from pigeons, with New York City subsampled (n=70). Similar to the DAPC on the full dataset, this graph recapitulates geography, showing separation of sampled collected in the northern part of the megacity (Boston and Providence) from samples collected in more southern parts of the megacity along the 1^st^ PC axis (x-axis) and New York City/Philadelphia samples separating from the Baltimore/Washington DC samples along the 2^nd^ PC axis (y-axis).


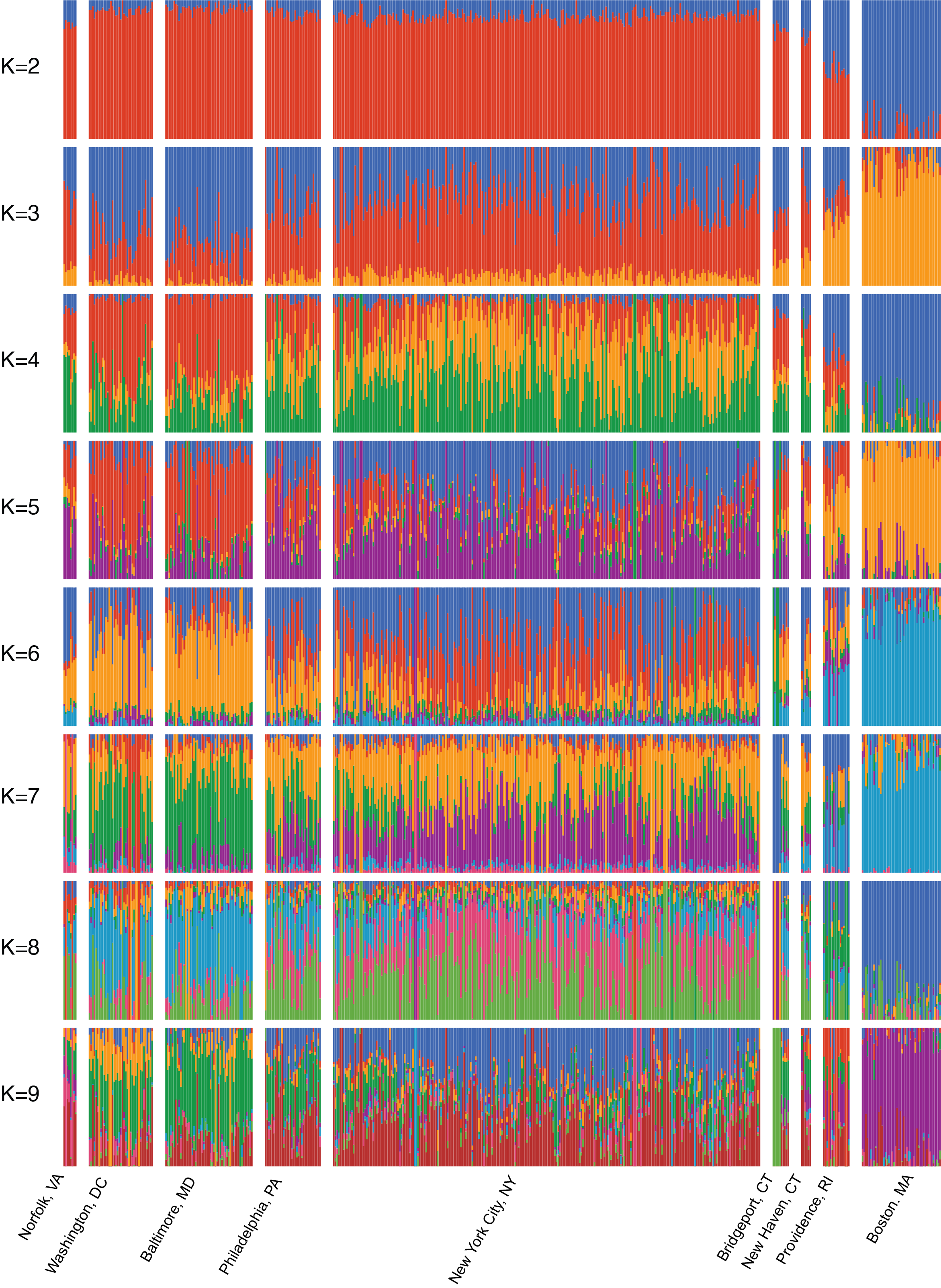


Figure S3: ADMIXTURE bar plots at K=2 through K=9. Samples from Boston consistently cluster as different from samples collected in more southern cities. Cross-validation indicated that K=2 is the most well supported K value.


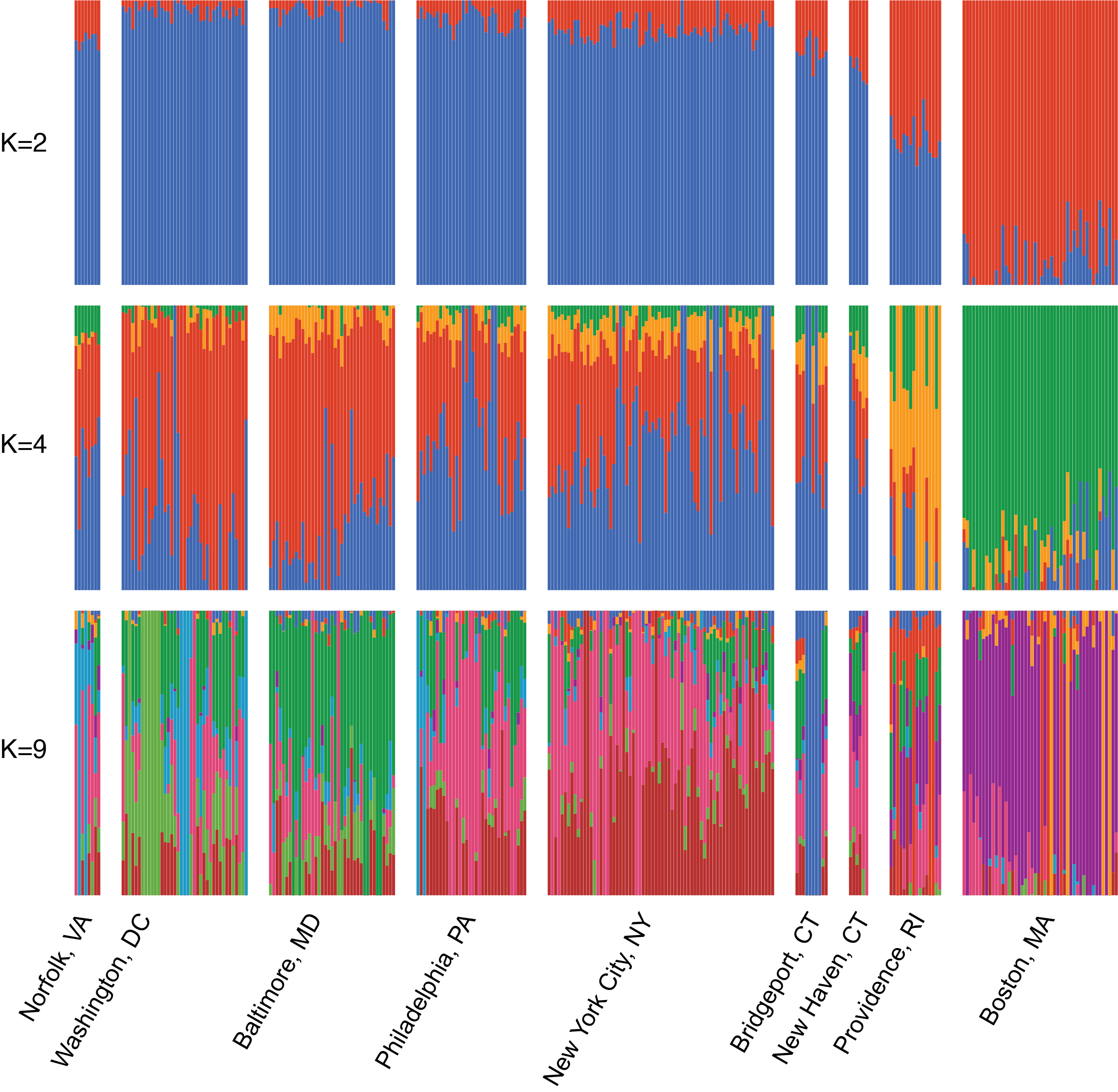


Figure S4: ADMIXTURE bar plots at K=2, K=4, and K=9 with a subsample of pigeons from New York City (n=70). Samples from Boston consistently cluster as different from samples collected in more southern cities. Cross-validation indicated that K=2 is the most well supported K value.

Table S1. Fixation index (F_ST_) comparing pigeons collected in each of the nine cities that were sampled. Sample sizes for each of the populations are as follows: Norfolk, VA, n=8; Washington, DC, n=39; Baltimore, MD, n=53; Philadelphia, PA, n=34; New York City, NY, n=259; Bridgeport, CT, n=10; New Haven, CT, n=6; Providence, RI, n=16; Boston, MA, n=48.

|  | Washington, DC | Baltimore, MD | Philadelphia, PA | New York City, NY | Bridgeport, CT | New Haven, CT | Providence, RI | Boston, MA |
| --- | --- | --- | --- | --- | --- | --- | --- | --- |
| Norfolk, VA | 0.015 | 0.011 | 0.017 | 0.003 | 0.040 | 0.047 | 0.030 | 0.017 |
| Washington, DC |  | 0.008 | 0.010 | 0.003 | 0.016 | 0.015 | 0.017 | 0.018 |
| Baltimore, MD |  |  | 0.008 | 0.003 | 0.012 | 0.011 | 0.013 | 0.018 |
| Philadelphia, PA |  |  |  | 0.003 | 0.018 | 0.017 | 0.018 | 0.017 |
| New York City, NY |  |  |  |  | 0.003 | 0.002 | 0.004 | 0.008 |
| Bridgeport, CT |  |  |  |  |  | 0.040 | 0.028 | 0.018 |
| New Haven, CT |  |  |  |  |  |  | 0.029 | 0.015 |
| Providence, RI |  |  |  |  |  |  |  | 0.014 |

Table S2. Summary of population genetics statistics calculated by STACKS for nucleotide positions that were polymorphic in at least one of the nine populations sampled. Number of individuals sequenced in each population (n) is shown along with average number of individuals genotyped at each locus (mean per locus). The average frequency of the major allele (P), mean observed heterozygosity (H_o_), mean expected heterozygosity (H_e_), mean nucleotide diversity (π), and mean inbreeding coefficient (F_IS_) for each population are also shown.

| collection location | n | mean per locus | P | H_o_ | H_e_ | π | F_IS_ |
| --- | --- | --- | --- | --- | --- | --- | --- |
| Norfolk, VA | 8 | 7.4 | 0.873 | 0.195 | 0.187 | 0.201 | 0.012 |
| Washington, DC | 39 | 36.1 | 0.864 | 0.199 | 0.209 | 0.212 | 0.041 |
| Baltimore, MD | 53 | 50.1 | 0.862 | 0.205 | 0.213 | 0.215 | 0.031 |
| Philadelphia, PA | 34 | 29.6 | 0.867 | 0.196 | 0.204 | 0.207 | 0.035 |
| New York City, NY | 259 | 230.4 | 0.867 | 0.195 | 0.206 | 0.207 | 0.036 |
| Bridgeport, CT | 10 | 9.6 | 0.869 | 0.204 | 0.197 | 0.208 | 0.010 |
| New Haven, CT | 6 | 5.7 | 0.872 | 0.189 | 0.187 | 0.205 | 0.038 |
| Providence, RI | 16 | 15.3 | 0.863 | 0.207 | 0.208 | 0.215 | 0.024 |
| Boston, MA | 48 | 44.9 | 0.864 | 0.198 | 0.208 | 0.210 | 0.039 |

Table S3. NeEstimator results showing estimated effective population size. Number of individuals sequenced in each population (n) is shown along with the NeEstimator results from five different runs using 10,000 randomly sampled SNPs for each run and the mean across the five runs ($\mathbf{x̄} INCLUDEPICTURE "https://wikimedia.org/api/rest\_v1/media/math/render/svg/466e03e1c9533b4dab1b9949dad393883f385d80" \backslash* MERGEFORMATINET$).

| **collection location** | **n** | **Ne test 1** | **Ne test 2** | **Ne test 3** | **Ne test 4** | **Ne test 5** | $\boldsymbol{x̄} INCLUDEPICTURE "https://wikimedia.org/api/rest\_v1/media/math/render/svg/466e03e1c9533b4dab1b9949dad393883f385d80" \backslash* MERGEFORMATINET$ |
| --- | --- | --- | --- | --- | --- | --- | --- |
| **Norfolk, VA** | 8 | Infinite | Infinite | Infinite | Infinite | Infinite |  |
| **Washington, DC** | 39 | 405.8 | 392.4 | 401.0 | 419.4 | 426.0 | 408.92 |
| **Baltimore, MD** | 53 | 957.9 | 842.4 | 907.2 | 840.6 | 849.5 | 879.52 |
| **Philadelphia, PA** | 34 | 531.7 | 523.1 | 536.7 | 495.1 | 534.0 | 524.12 |
| **New York City, NY** | 259 | 2492.7 | 2440.3 | 2502.4 | 2533.6 | 2534.9 | 2500.78 |
| **Bridgeport, CT** | 10 | 46.1 | 43.1 | 43.0 | 47.3 | 42.6 | 44.42 |
| **New Haven, CT** | 6 | Infinite | Infinite | Infinite | Infinite | Infinite |  |
| **Providence, RI** | 16 | 117.6 | 107.4 | 117.0 | 109.0 | 108.4 | 111.88 |
| **Boston, MA** | 48 | 428.9 | 454.1 | 416.7 | 420.8 | 427.7 | 429.64 |

| **collection location** | **n** | **Ne test 1** | **Ne test 2** | **Ne test 3** | **Ne test 4** | **Ne test 5** | $\boldsymbol{x̄} INCLUDEPICTURE "https://wikimedia.org/api/rest\_v1/media/math/render/svg/466e03e1c9533b4dab1b9949dad393883f385d80" \backslash* MERGEFORMATINET$ |
| --- | --- | --- | --- | --- | --- | --- | --- |
| **Southern Cluster**  **Norfolk, VA**  **Washington, DC**  **Baltimore, MD**  **Philadelphia, PA**  **New York City, NY**  **Bridgeport, CT**  **NewHaven, CT** | 409 | 2925.4 | 2921.6 | 2949.0 | 2905.8 | 2933.1 | 2927.0 |
| **Northern Cluster**  **Providence, RI**  **Boston, MA** | 64 | 393.4 | 390.1 | 395.7 | 423.2 | 393.4 | 399.2 |
